# Widespread support for a global species list with a formal governance system

**DOI:** 10.1073/pnas.2306899120

**Published:** 2023-10-30

**Authors:** Aaron M. Lien, Olaf Banki, Saroj K. Barik, John S. Buckeridge, Les Christidis, María Marta Cigliano, Stijn Conix, Mark John Costello, Donald Hobern, Paul M. Kirk, Andreas Kroh, Narelle Montgomery, Svetlana Nikolaeva, Thomas M. Orrell, Richard L. Pyle, Lauren Raz, Kevin Thiele, Scott A. Thomson, Peter Paul van Dijk, Nina Wambiji, Anthony Whalen, Frank E. Zachos, Zhi-Qiang Zhang, Stephen T. Garnett

**Affiliations:** ^a^School of Natural Resources and the Environment, College of Agriculture, Life and Environmental Sciences, University of Arizona, Tucson, AZ 85721; ^b^Species 2000, Naturalis, Leiden 2300 RA, Netherlands; ^c^Department of Botany, North-Eastern Hill University, Shillong 793022, India; ^d^Earth and Oceanic Systems Group, RMIT University, Melbourne, VIC 3001, Australia; ^e^Southern Cross University, Coffs Harbour, NSW 2450, Australia; ^f^Museo de La Plata, Centro de Estudios Parasitológicos y de Vectores - Consejo Nacional de Investigaciones Científicas y Técnicas, Universidad Nacional de La Plata, La Plata B1900FWA, Argentina; ^g^Institut Supérieur de Philosophie, Université Catholique de Louvain, Ottignies-Louvain-La-Neuve 1348, Belgium; ^h^Faculty of Biosciences and Aquaculture, Nord Universitet, Bodø 8049, Norway; ^i^Atlas of Living Australia, Commonwealth Scientific and Industrial Research Organization Black Mountain, Canberra, ACT 2601, Australia; ^j^Royal Botanic Gardens Kew, Richmond, London TW9 3AB, United Kingdom; ^k^Natural History Museum Vienna, Vienna 1010, Austria; ^l^Department of Climate Change, Energy, the Environment and Water, Canberra ACT 2600, Australia; ^m^Sessional Committee, Scientific Council, Convention in the Convention on the Conservation of Migratory Species of Wild Animals, Bonn 53113, Germany; ^n^Department of Earth Sciences, The Natural History Museum, London SW7 5BD, United Kingdom; ^o^Laboratory of Molluscs, Borissiak Paleontological Institute, Russian Academy of Sciences, Moscow 117647, Russia; ^p^Research Laboratory of Stratigraphy of Oil-and-Gas Bearing Reservoirs, Kazan Federal University, Kazan 420008, Russia; ^q^Informatics and Data Science Center, Smithsonian Institution, National Museum of Natural History, Washington, DC 20013; ^r^Bernice Pauahi Bishop Museum, Honolulu, HI 96817; ^s^Instituto de Ciencias Naturales, Universidad Nacional de Colombia, Bogotá 111321, Colombia; ^t^Research School of Biology, Australian National University, Canberra ACT 2600, Australia; ^u^Centro de Estudos dos Quelônios da Amazônia, Manaus 69055-010, Brazil; ^v^Research Institute for the Environment and Livelihoods, Charles Darwin University, Darwin NT 0909, Australia; ^w^Re:wild, Austin, TX 78767; ^x^Kenya Marine and Fisheries Research Institute, Mombasa 80100, Kenya; ^y^National Research Collections Australia, Commonwealth Scientific and Industrial Research Organisation, Canberra ACT 2601, Australia; ^z^Department of Evolutionary Biology, University of Vienna, Vienna 1030, Austria; ^aa^Department of Genetics, University of the Free State, Bloemfontein 9301, South Africa; ^bb^New Zealand Arthropod Collection, Manaaki Whenua–Landcare Research, St Johns, Auckland 1072, New Zealand; ^cc^Centre for Biodiversity and Biosecurity, School of Biological Sciences, The University of Auckland 1010, Auckland, New Zealand

**Keywords:** taxonomists survey, taxonomy, classification, species lists, governance

## Abstract

Taxonomic data are a scientific common. Unlike nomenclature, which has strong governance institutions, there are currently no generally accepted governance institutions for the compilation of taxonomic data into an accepted global list. This gap results in challenges for conservation, ecological research, policymaking, international trade, and other areas of scientific and societal importance. Consensus on a global list and its management requires effective governance and standards, including agreed mechanisms for choosing among competing taxonomies and partial lists. However, governance frameworks are currently lacking, and a call for governance in 2017 generated critical responses. Any governance system to which compliance is voluntary requires a high level of legitimacy and credibility among those by and for whom it is created. Legitimacy and credibility, in turn, require adequate and credible consultation. Here, we report on the results of a global survey of taxonomists, scientists from other disciplines, and users of taxonomy designed to assess views and test ideas for a new system of taxonomic list governance. We found a surprisingly high degree of agreement on the need for a global list of accepted species and their names, and consistent views on what such a list should provide to users and how it should be governed. The survey suggests that consensus on a mechanism to create, manage, and govern a single widely accepted list of all the world’s species is achievable. This finding was unexpected given past controversies about the merits of list governance.

Species lists and the taxonomic decisions embedded in them play a critical role in our fundamental understanding of the natural world, policymaking about species management, conservation biology and the management of threatened and endangered species, and many other decisions of scientific and societal importance (1–3). Despite this, while there are well-established governance systems in place for nomenclature, there is no similar system for the systematic aggregation and maintenance of species names and current classifications into a globally accepted list to meet scientific and societal needs (4). Creation of such a governance system would require engagement with a diverse and globally distributed community of taxonomists and other users of taxonomy. Species are described, named, and published by taxonomists in accordance with the rules of established nomenclatural codes. This process provides not only an important foundational structure to taxonomy but also a challenge when there are competing taxonomic concepts and/or alternative names for individual species.

In 2017, Garnett and Christidis published a short commentary in *Nature* that identified a mismatch in the ways policy and science deal with species (5). Policy assumes that species are unambiguously definable, discrete, fixed entities that can be readily and straightforwardly listed for protection or management. However, in the sciences of species delimitation and classification (i.e., taxonomy and systematics), species are hypotheses that are mutable and contestable in the face of new knowledge and are often based on subjective interpretations of existing knowledge (6, 7). This is because taxonomy is essentially a two-step enterprise—first, biodiversity and groups are quantified, described, and delimited by means of the best scientific methodology available, and then these results are translated into names and ranks. While the first step is strictly scientific and produces testable hypotheses, the second additionally depends on executive decisions about where to draw the line (e.g., between subspecies and species), and these decisions necessarily depend on subjective preferences such as one’s preferred species concept or the nature of discordance among different data types.

Users would benefit from an agreed, unitary, relatively stable list of all the world’s species. Such a list would greatly aid global biodiversity conservation, management, information retrieval, and communication (5). With mass extinctions inevitable without concerted global action, efficient communication about taxonomy can avoid wasteful and distracting debates (8). For example, a single authoritative global species list would have in-built quality control protecting users from the confusion resulting from names created through what has been called taxonomic vandalism (see ref. 8 for details and the role that trusted species lists play in herpetology). Multiple lists also mean extra, and often duplicated, effort when taxonomic resources are scarce. In addition, it has been shown that many quantitative analyses in ecology and evolution that rely on species richness (i.e., numbers of different species in a given taxon or geographic region) are critically affected by different underlying taxonomic approaches (1, 2). However, achieving such a global list depends on the work of many taxonomists, who may be working with different species taxonomies and would need convincing to agree readily on a unified list of species in any given taxonomic group. Achieving general agreement on basic principles of governance among the diverse membership of the taxonomic community and those who use taxonomic research is a substantial challenge.

The partial solution proposed by Garnett and Christidis, that a governance body should be established to define the process for how species are accepted and how differing opinions are managed, sparked concern among many taxonomists (9–14). Some argued such a body would introduce unnecessary bureaucracy to a fundamentally scientific process. With many species still awaiting description and new techniques such as environmental deoxyribonucleic acid (eDNA), metabarcoding, and other genetic approaches increasing the rate of discovery of new taxa, there was a fear that barriers introduced by a governance system could slow progress unnecessarily (9). It was also argued that peer review was already providing a basic form of governance by identifying good science, weeding out poor science, and reconciling scientific disputes through the churn of scientific debate and discovery (10). However, it is this very churn that causes problems for policymakers, who do not have the scientific expertise themselves to evaluate competing claims, nor the wherewithal to maintain a comprehensive and contemporary knowledge of the literature. Peer review also does little to resolve legitimate scientific differences, nor is all taxonomic information peer reviewed.

Others argued that a governance system for taxonomic lists is a threat to fundamental scientific freedom (11, 13). Debate and dissent are fundamental to scientific process, producing new insights and knowledge by challenging current understandings of the world. Taxonomy is not only an observational science but is also hypothesis-driven and a means of abstracting the complexity of the natural world into more systematic and easily understood components (7). In conducting this work, alternative hypotheses about how to organize taxa inevitably and legitimately arise.

Spurred by this debate, and to overcome differences in understanding about the meaning and goals of list governance, in 2020, a multinational group of taxonomists, scientists from other disciplines of biology, and users of taxonomy formed, under the auspices of the International Union for Biological Sciences, a Global Species List Working Group (GSLWG). Many of the members of the GSLWG were already engaged in vigorous debate about the value of species list governance; the working group provided a constructive forum to discuss different viewpoints and identify if common ground existed in favor of advancing a process to establish a governance system for producing and maintaining a global taxonomic list. The GSLWG collaboration resulted in a set of principles for the governance of taxonomic lists that would not interfere with the science behind taxonomy and would ease decision making in policy, conservation, and other arenas (15). The GSLWG also explored the processes and practices of taxonomy and the management of a global list of accepted species in detail (7–8, 16–19).

Any governance system to which compliance is voluntary requires a high level of legitimacy and credibility among those for whom it is created (20, 21). Legitimacy and credibility, in turn, require adequate and credible consultation, for example, through community engagement to develop shared norms and inclusive governance institutions (22, 23). As a scientific common, basic principles of self-organization and influence on the structure of governance institutions are important for the acceptance of a new governance system and for the likelihood of governance overcoming collective action barriers (20). While the GSLWG is composed of scientists and users with a range of views on list governance, it is also a self-selected group and may not represent the opinions of the community of taxonomists, other scientists, or users of taxonomic information. To address this limitation, we conducted a first-of-its-kind survey designed to gather the opinions of taxonomists, scientists from related disciplines, and users of taxonomic information that aimed to assess current preferences for taxonomic list governance and content. This survey was conducted in Spring 2022 and consisted of 42 questions addressing overall support for a governance system, the basic desired characteristics for a governance system should one be developed, and criteria and information requirements for inclusion of species on a global list (*SI Appendix*).

The responses demonstrated remarkable agreement across stakeholder groups that a single global list is needed. Respondents also agreed on the taxonomic information that should be included in a global species list and on the basic criteria for inclusion of taxa on such a list. Some of the consistency was unexpected. For example, respondents who identified as users were as enthusiastic about attribution of names with dates and authors, as were taxonomists. The results of the survey provide a clear path for the development of a governance mechanism for achieving maximum agreement on a global list of accepted species. Such a governance system would benefit both taxonomists and user communities.

## Respondents Represent a Broad Cross-Section of the Scientific and User Community

We received 1,134 valid responses (82% of the sample of total responses; see *Materials and Methods* for details). Of these, 560 were taxonomists (49%), 409 were scientists in other disciplines (36%), and 50 were users of taxonomic information (4%). An additional 82 selected both taxonomist and scientist (7%) and 33 selected user plus at least one other category (3%) (*SI Appendix*, Table S1). Among taxonomists, 86% were involved in species descriptions, 64% in phylogeny, and 51% in development of species checklists. Of the 50% who identified their area of specialization, 16% concentrated on vertebrate taxonomy, 51% on invertebrates, and 29% on plants or fungi. Other scientists included ecologists (55%), conservation biologists (40%), evolutionary biologists (25%), biogeographers (24%), and bioinformaticians (12%). Most users of taxonomic information were engaged in conservation work (40%), education (16%), or trade (8%). Responses were received from 74 countries, with the largest numbers from Germany (190), the United States of America (110), Australia (85), and India (83).

## Support for a Global List and its Contents

**Fig. 1.**
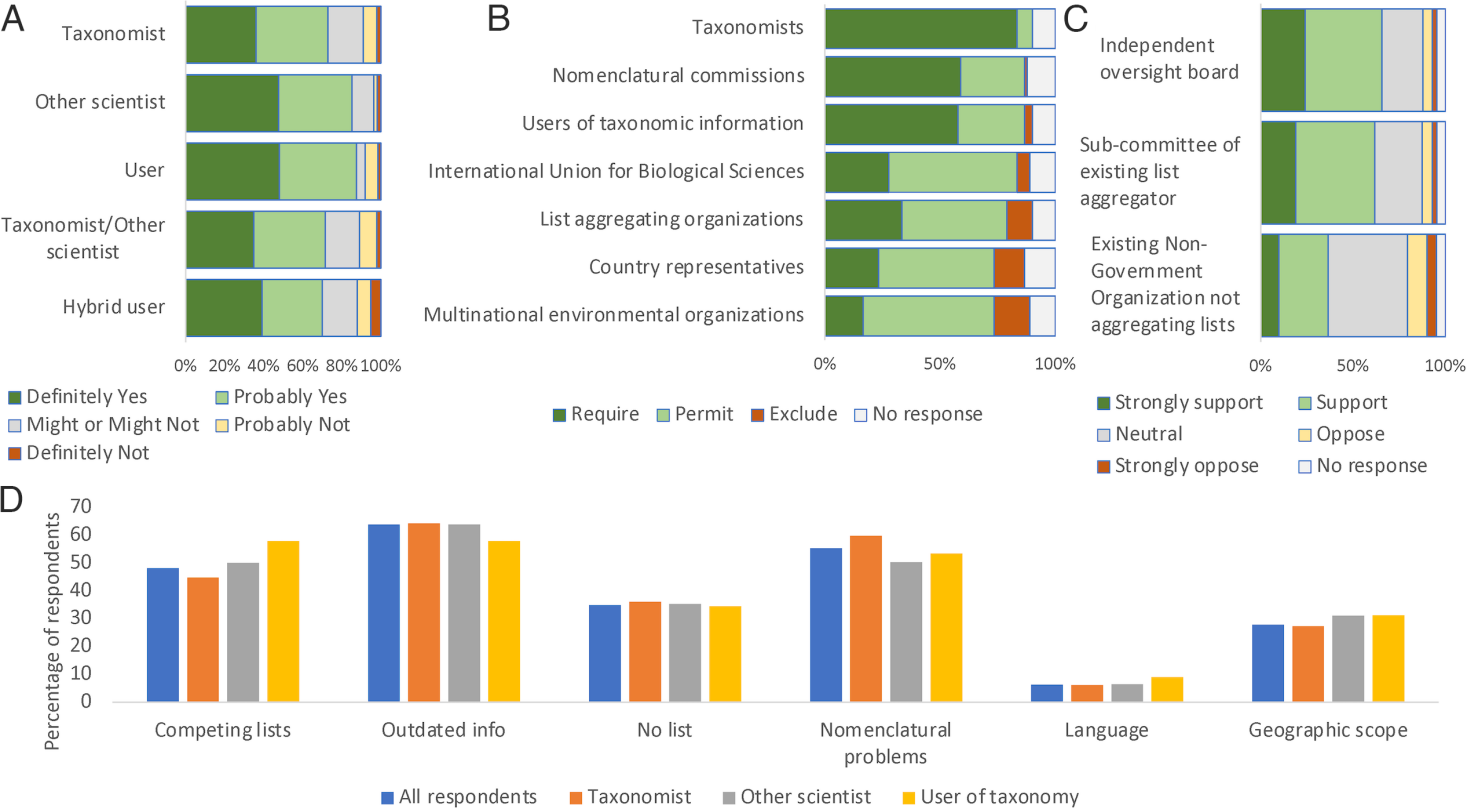
Survey results showing (*A*) generally strong agreement that there would be a net benefit to creation of a single global species list across all respondent groups; (*B*) overall preferences for representation on an oversight board; (*C*) overall preferences for oversight options of an independent board, a committee of an existing organization, or an NGO; and (*D*) common problems encountered by respondents, including competing lists, outdated information, and the absence of lists.

A high proportion of respondents (77%; Fig. 1) agreed that a unified global list of all life forms would be a net benefit, with the greatest support among users of such lists (88%). Scientists who were not taxonomists were also strongly supportive (85%). Even among taxonomists, who might be expected to be most skeptical of such a list, support was high (73%). About half of the respondents in all categories use taxonomic information contained in species lists on specific taxa or in national and/or regional lists frequently or on a daily basis. The problems most frequently encountered by respondents in finding and using taxonomic information, which a global list could potentially help overcome, included: outdated lists (64%), nomenclatural problems (55%), competing lists (48%), and a lack of lists for specific taxa (35%). Geographic scope of lists and the language used were selected relatively infrequently. For taxonomists who identified their area of specialization (n = 548), there was a marked disparity between vertebrate, invertebrate, and plant taxonomists. Nearly all vertebrate taxonomists reported problems with nomenclature and outdated or competing lists, which was about double the proportion for invertebrate taxonomists and over three times the proportion for plant taxonomists. In written feedback, these problems were further emphasized, as many respondents complained of data that were conflicting or difficult to access for different species groups, the slow pace at which existing species lists are updated, particularly those in print form, and, importantly, an inability to judge the provenance of the data included in existing lists. This emphasizes the overall importance of a global species list as a critical source of information and highlights the challenges that can arise when there are conflicting lists. None of the 87 respondents who did not think there would be a benefit from having a global list (8% total; taxonomists 9%, other scientists 4%, and users 8%) provided any explanation of their reasoning.

The high level of agreement persisted across most types of information about species that could be included in a global list, including use of unique species identification numbers, genus-level classification, classification above genus-level, author(s) of the treatment and nomenclature, and statements about uncertainty in taxonomic classification status. Stakeholders were more ambivalent about the inclusion of vernacular names of species and the need for a user moderation system to, for example, allow for user comments and discussion, though these topics also attracted support from more than half the respondents. This type of information represents basic data standards for inclusion of a species in a global list and is not list governance per se. Efforts such as the International Plant Names Index and ChecklistBank already perform a similar function of compiling data about species. Rather, list governance is the system of rules that are used to evaluate and aggregate taxa-specific lists into a larger global list, who is empowered to develop and modify these rules, and who provides the oversight and implementation of the rules over time.

## Governance of a Global Species List

The core challenge of developing an accepted global list of species is not the information it contains or its technical management, as is evident by the broad agreement across respondents and the existence of dynamic online databases like the World Register of Marine Species (WoRMS) (24, 25) but in how the governance of such a list should be organized. Governance, broadly construed, is a system of organization that guides the actions of the governed (26). A governance system for a global list of species raises numerous questions: How is the list generated; what data quality standards must be met for the inclusion of a species on the global list; who decides; and how are disputes between competing lists resolved? Just as there was strong support for creation of a single global list, there was much common ground for governance arrangements that support the development and maintenance of a global species list.

Of the many options that exist for general administration of a global list (18), we presented respondents with three broad options: i) an independent oversight board or commission; ii) a subcommittee of an existing aggregator such as the Catalogue of Life (https://www.catalogueoflife.org/); or iii) another existing nongovernmental organization (NGO) not involved in species list aggregation, such as the International Union for Biological Sciences. Support was similar for the first two options (72% and 68% respectively support or strongly support; Fig. 1) but weak (40%) for the third. All respondents considered that taxonomists should be required as members (95% across all respondents) and there was also general agreement that nomenclatural commissions (e.g., International Commission on Zoological Nomenclature, International Association for Plant Taxonomy) should also be represented (69%). Majorities of nontaxonomist scientists and users of taxonomic information also favored required representation from users (56% and 63%, respectively), but for taxonomists, the participation of users was considered acceptable rather than essential (54% agreed with permitting user representation, but only 38% thought it should be required). All respondents favored permitting representation for country representatives, NGOs, aggregators, and the International Union for Biological Sciences (IUBS). A substantial majority of respondents agreed that any oversight body should reflect both the diversity of taxonomic fields (91%) and the geographic spread of taxonomists (72%), but less than half thought that quotas were needed for any category (37%).

The selection process for an oversight body could influence representation and ultimately the content of the list. For example, an open voting process could result in an oversight body dominated by taxonomists from the most-studied groups or by users of taxonomic information who are not themselves directly engaged in the work of taxonomic science. We presented respondents with three general options: i) taxonomic specialty groups should develop their own process for selection of representatives, ii) a nomination and voting process managed by the oversight body, or iii) self-selection. Small majorities of taxonomists (51%) and other scientists (53%) preferred that taxonomic specialty groups be allowed to develop their own process for selection (option 1), while a small majority of users preferred a nomination process (52%, option 2). There was scant support for self-selection across all groups (4%, option 3).

If a voting process were adopted for selection of representation to an oversight body, most respondents supported using involvement in the creation of taxonomic lists as a criterion for eligibility to vote (56% taxonomists; 59% other scientists; 50% users). A substantial proportion of taxonomists (50%) and other scientists (41%) also considered that voting eligibility should be constrained to people with five or more peer-reviewed publications in taxonomy (an indicator of a basic level of knowledge of taxonomic science). One publication was not seen as adequate by any group, nor was there support for a voting process that allowed self-selection, or anyone with a demonstrated interest in taxonomy to vote.

The final portion of the survey addressed opinions about the criteria for inclusion or exclusion of taxa from a global species list and preferred approaches to dispute resolution. Inclusion criteria go to the core purpose of a governance system and its institutional arrangements: they provide the rules that are followed by those with decision-making authority, regardless of who those decision-makers are or how they are selected. We presented respondents with 15 specific criteria for the determination of eligibility for inclusion (*SI Appendix*, Table S2), with questions focused primarily on understanding minimum standards for data quality, provenance, and transparency. Respondents across groups largely agreed that the most important criteria for inclusion and aggregation of taxon-specific lists into a single global list were that a) taxon-specific species lists are based solely on science and no other considerations (89%), b) the people who contributed to the development of taxon-specific lists should be acknowledged (92%), and c) taxon-specific lists should use open-access licensing (90%), such as a CC-BY license where users of the list must cite the source. There was also broad agreement that the criteria for differentiating species should be clearly stated (81%) and that there be a clear quality assessment process applied to taxon-specific lists before submission for inclusion in the global list (88%). Majorities agreed or strongly agreed with all the other criteria included in the survey but with larger minorities of respondents either neutral or in disagreement.

Dispute resolution is central to any governance system (26) and the conflicts inherent in competing lists were identified as a problem by many respondents. Respondents provided similar levels of support for either a) tasking an oversight body with development of and implementation of a dispute resolution process (40%) or b) an independent process, which was left undefined (41%). While more taxonomists favored an independent process (42% compared to 35% in favor of tasking the oversight body), resolution by the oversight body was favored by pluralities of other scientists (45%) and users (53%). Most of the 14% of respondents who provided written responses supported reference to all competing lists for maximum transparency, even if only one list is included in the final global list. Some also suggested tagging taxon groups for which multiple lists exist to encourage taxon-specific groups to seek agreement. An intrataxon dispute resolution process was also favored as a list inclusion criterion (73%).

## Building on Community Support to Advance List Governance

The responses to our survey point to taxonomic list governance arrangements that would satisfy a wide range of taxonomists, other scientists, and users and outline the characteristics of a taxonomic list of high quality. A large majority of respondents (77%) supported the development of a governance system designed to create and maintain a single list of life on Earth. This majority held across all categories of respondents, including taxonomists (73%), other scientists (85%), and users of taxonomic information (88%). Respondents preferred that an independent oversight board (72%) or a subcommittee of a current list aggregator (68%) made up of taxonomists representing a diversity of taxa and geographies, and nomenclatural commission representatives, administer the global list. Other potential members of the oversight body include civil society and users. Elections were the preferred method of selection of representatives to the oversight body, though opinions were mixed on how to constitute these elections.

The number and geographic spread of respondents and the breadth of taxonomic specialization they represented, as well as the high level of agreement among them, suggest that governance arrangements guided by the survey responses have the potential to gain a legitimacy, which would be difficult to attain for any separate governance system developed by a list aggregator on its own. There is a small risk that our survey approach, snowballing through the networks of the GSLWG, might have biased results, given that the GSLWG was assembled to debate and advance global species list governance. However, many of the lists used to distribute the survey were general membership lists for organizations, e.g., Gesellschaft für Biologische Systematik, the Network for Biological Systematics, the Scientific Council of the Convention on Migratory Species, World Flora Online Council, and many others (*SI Appendix*, Table S3), rather than personal contact lists of the authors. The diversity and number of respondents to the questionnaire provide evidence that the survey elicited responses from networks to which GSLWG members had no direct connection, and the earlier history of vigorous debate among GSLWG members (5, 13, 27) suggests that respondents with a diverse range of views were reached. Authors of papers contesting the idea of global list governance following publication of Garnett and Christidis’ commentary (5) were also directly invited to respond.

Indeed, the process developed by the GSLWG is consistent with the ideals of the scientific process. Garnett and Christidis’ (5) suggestion that a governance system for taxonomic lists was needed was, as noted in the introduction to this paper, met with skepticism and was widely debated in the scientific literature (9–11, 13). In response, instead of abandoning the idea, the original arguments were refined and improved taking early criticism into account and with the help of a much larger group of scientists from taxonomy and other fields, including many of those who published critical critiques of Garnett and Christidis’ (5) commentary (7–8, 12, 15–19, 27). The results of the survey show that support for list governance in the taxonomic community, and the wider community of scientists and users of taxonomic information, is now widespread. This change may be the product of revision and refinement of initial concepts into a set of principles that directly respond to community concerns (15), increased comfort with the concept of governance after extensive debate, or an indication that skepticism of list governance was perhaps not as widespread as initially thought. The lack of other surveys of the taxonomic community limits our ability to determine the cause of the shift this survey appears to indicate. Regardless, instead of debating the need for governance, the discussion should now shift to how such a system could function. While much work remains, it appears the time for the establishment of governance institutions to support the development of a single list of species on Earth has come.

Creating lists of accepted species and their names is a core activity for taxonomists. Assembly of such lists to create a single global list of accepted species requires substantial informatic skill and infrastructure, especially given that over half of all species names are synonyms (4, 28). Such capacity has been developed by the Catalogue of Life [partnering with the Global Biodiversity Information Facility (GBIF)], which has worked since 2001 to collate species lists and currently includes names for over 2 million species (data deriving from environmental- or taxonomy-specific global lists like the WoRMS, World Plants, Integrated Taxonomic Information System, Species Fungorum Plus, Systema Dipterorum, World Checklist of Vascular Plants, Global Lepidoptera Index, Species Files, and many others) (29). To date, its efforts have been driven from within the taxonomic community and users have not been explicitly included in its governance (apart from taxonomists being users themselves). Partly for this reason, species-focused multinational environmental agreements (e.g., Convention on International Trade in Endangered Species, Convention on Migratory Species), government agencies, research institutes, and organizations such as the International Union for Conservation of Nature use a diverse range of sometimes mutually inconsistent taxonomic lists, despite the resultant confusion and inefficiency.

Given the level of support for rules to assess list quality and for list aggregation, the primary task now will be the creation of an oversight body to perform these tasks. While there is broad support for developing either a new, independent governing body, or building one within the organizational structure of an existing aggregator of taxonomic lists such as Catalogue of Life, eventually a choice will need to be made. The Catalogue of Life maintains a data infrastructure that supports most of the information needs desired by respondents, has a stated goal of creating an “authoritative list of the world’s species” (17), and has an established relationship with the GBIF, the world’s largest database of biodiversity information. However, creation of a governance structure within an existing organization is not without risk. Organizations have reputations that vary across a community as large and diverse as taxonomists, other scientists who use taxonomy, and users of taxonomic information. As a result, some may be discouraged from contributing to a global list depending on the host entity, and users of taxonomic information may have higher levels of trust for certain organizations over others. Whether the governance body is established as a new entity or within another body, it would need to secure new resources to be effective and would need sufficient recognition and authority within the taxonomic community to achieve its goals. Such details will require additional consultation across stakeholder groups once details of a possible species list governance structure are available (18).

Similarly, a key challenge for progress toward a broadly recognized, single global list of the world’s species is reconciliation or resolution of incompatibilities between competing lists. There was no clear agreement on the best approach to this challenge or if it is even desirable to reconcile competing lists. While many respondents considered competing lists a problem, others argued that multiple lists for a given taxon should be available as a legitimate part of the scientific process. Such transparency can lead to subsequent collaboration and integration between overlapping list compilers, as has been the experience in developing WoRMS (5, 24). If a global species list is to lead to more consistent protection and management of species at international and national levels, an approach to reconciling these perspectives is necessary. In its deliberations, the GSLWG, in order to adhere to core principles of scientific freedom (15), has offered a governance model that establishes basic procedural standards for the development of taxa-specific lists proposed for aggregation into a single global list (17, 19). Under this model, the global species list oversight body does not engage in taxonomic decisions. The creators of taxa-specific lists engage with their own community to develop lists, reconcile differences in taxonomic treatments, and overcome conflict. Success will likely depend on the development or selection of a trusted entity to lead this process of developing and implementing a system of global list governance. While far from assured, given the broad agreement across respondents to our survey, we are optimistic that any obstacles can be overcome.

Much work is still needed to build from the broad agreement on governance principles identified in this survey. Because the governance system would be voluntary, it will only be successful if the community of taxonomists, scientists in other disciplines that contribute to and use taxonomy, and users of taxonomic information in other fields continue their support. Going forward, having gauged the legitimacy of a taxonomic list governance body, the GSLWG is now working with the Catalogue of Life to investigate alternative governance models and metrics for assessing list governance quality in detail. Catalogue of Life partners with GBIF to create ChecklistBank and deliver the Catalogue of Life Checklist. ChecklistBank is an open respository for taxonomic checklists including a broad range of tools for quality control, derivation of new checklist datasets, and flexible reuse of list data. The Catalogue of Life Checklist and other ChecklistBank datasets help improve the consistency of GBIF’s biodiversity data records. However, it is premature to suggest what entity might manage a governance system until all options, including capacity, are fully explored. The results of this process will serve as the basis for further consultation and engagement with the broader taxonomic community with the goal of developing governance standards that lead to the creation and maintenance of a global species list.

## Materials and Methods

We conducted the survey on list governance online to reach as broad an audience as possible (*SI Appendix*, Table S1) (30). Survey questions aimed to test the acceptability or desirability of aspects of list governance raised in earlier publications to taxonomists, other scientists, and users of taxonomic information (7–8, 15–19). The survey was preregistered on the Open Science Framework (OSF; https://doi.org/10.17605/OSF.IO/7DFWT). The Charles Darwin University Human Research Ethics Committee reviewed and approved the survey under protocol number H22012. Informed consent was conducted electronically at the beginning of the survey; the purpose of the survey was described, along with expected time commitment, risks, and expectations for confidentiality of responses. Respondents were explicitly asked a question to provide consent before the start of the survey and only began the survey if they responded affirmatively. If consent was declined, the survey ended with no additional questions or data collected. Translations from English were available in simplified Chinese, French, Italian, Portuguese, and Spanish. The survey was distributed by members of the GSLWG to individuals and groups within their professional networks and using snowball methods. This included people working as taxonomists, scientists in other fields who use taxonomic information, and organizations that are involved in the integration of taxonomic information or lists such as the Catalogue of Life. Direct recipients were also encouraged to distribute the survey further through their own professional networks. While it was possible to track recipients directly contacted by members of the GSLWG, we did not have direct access to many of the mailing lists to which the survey was sent by these recipients and therefore do not know the number of individuals these lists reached or the number of duplicate invitations. As a result of using the broad professional networks of the GSLWG and the mailing lists of other organizations, we expect we reached a wide audience of taxonomists, other scientists, and users of taxonomic information.

Recipients of the survey were asked to respond only if they self-identified as a taxonomist or a user of taxonomic lists, such as scientists in related disciplines or disciplines that rely on taxonomic information, NGOs, or those responsible for national or international taxonomic lists. Respondents could then identify themselves in the survey as 1) a taxonomist, 2) a scientist in another discipline, 3) a user of taxonomic information or 4) “other”; multiple categories could be selected. Those who listed themselves as other provided notes on their interest in taxonomic lists, on the basis of which all could be allocated during data analysis to one or more of the defined categories. The survey was available to respondents for approximately 1 mo beginning in mid-April 2022. During this 1-mo period, one invitation to participate and one reminder to participate was sent to the professional networks of the GSLWG. As noted, it is likely that some recipients received the initial invitation, the reminder, or both more than once because of overlaps between the networks of GSLWG members and redistribution by recipients to their own networks. Responses received within the month period were considered valid based on level of completion (i.e., >50% of questions answered).

After 1 mo, we closed the survey to further responses and began data analysis. Surveys that were less than 50% complete were excluded from consideration, resulting in a total of 1,134 valid responses. Responses were translated from languages other than English into English using Google Translate or by a native speaker in the GSLWG. Respondents who selected “Other” to describe their professional role in taxonomy were reviewed and reclassified as appropriate based on the information they provided in response to the request for a qualitative description along with the selection of other. Most people who selected other fitted easily within the category of “taxonomist” or “other scientist”. This classification was conducted by authors Garnett and Lien. Textual data on the studied organism groups provided by the respondents was assessed to discover more about the taxonomic scope of the participants. This assessment was conducted by the author Kroh.

The survey was exploratory in nature; we did not have predetermined hypotheses. Our goals were to understand the general viewpoints of taxonomists, other scientists, and users of taxonomic information towards the development of a governance system for taxonomic lists. We began our analysis by summarizing the data and conducting basic cross tabulations and Chi-Squared tests of the results. Given the consistency of responses within and between groups of survey respondents, additional analysis to interpret the results was deemed unnecessary.

## Supplementary Material

Appendix 01 (PDF)

## Data Availability

Anonymized survey data have been deposited in Governance of Taxonomic Lists Survey (DOI: 10.17605/OSF.IO/TZ7RA).
