# Supplementary material for: Widespread support for a global species list with a formal governance system
Source: Proc Natl Acad Sci U S A. 2023 Oct 30;120(45):e2306899120. doi: 10.1073/pnas.2306899120 (PMC10636331; doi:10.1073/pnas.2306899120)
Supplement: Supplementary file 1 — Appendix 01 (PDF) [file pnas.2306899120.sapp.pdf]

Supporting Information Appendix

Table 1: Percentage of respondents (n=1134) in each category and breakdown of self-reported professional activities within each category. Percentages within categories may exceed 100% because respondents were allowed to select more than one option.

Respondent Type	Percentage	Respondent Type	Percentage
Taxonomist	49	Other – Taxonomy	18
Species description	86	Ecology	55
Phylogeny	64	Biogeography	43
Checklist development	51	Paleontology	5
Other	29	Evolutionary Biology	41
Other Scientist	36	Microbiology	9
Ecology	55	Conservation biology	41
Biogeography	24	Bioinformatics	16
Paleontology	5	Other – Other Scientist	20
Evolutionary Biology	25	Hybrid Users	3
Microbiology	4	Species description	27
Conservation biology	40	Phylogeny	30
Bioinformatics	12	Checklist development	36
Other	29	Other – Taxonomy	21
Users of Taxonomy	4	Ecology	30
Conservation	40	Biogeography	15
Trade	8	Paleontology	12
Education	16	Evolutionary Biology	18
Other	16	Microbiology	9
Taxonomist/Other scientist	7	Conservation biology	42
Species description	63	Bioinformatics	24
Phylogeny	57	Other – Other Scientist	21
Checklist development	52		

Table 2. Candidate criteria for determination of eligibility for inclusion in a global list.

**Criteria**	
Annotations for user engagement	Non-Linnaean names
Classification above genus-level	Peer review
Confidence in taxonomic status	Recently extinct taxa
Fossil taxa	Recognition for list-creators
Genus-level classification	Synonyms of taxa
Geographical distribution	Treatment author, with citation
Images	Type-specimen information
Infraspecific taxa	Unique, persistent identifier
Nomenclatural author, with date	Vernacular names
Nomenclatural code	Version history

Table 3. Partial list of organizations that directly received notice of survey availability.

All authors on Thomson et al. 2018
All signatories to Wüster et al. 2021 (464)
Australian Society of Herpetologists
Catálogo Taxanômico da Fauna do Brasil
The Crustacean Society
Entomological Society of Argentina
Gesellschaft für Biologische Systematik
International Association for Biological Oceanography
IUCN Tortoise and Freshwater Turtle Specialist Group
Kenyan Chapter of the West Indian Ocean Marine Science Association
Museum of Natural History of the National University of La Plata
National Museum of Natural History, Buenos Aires
Network for Biological Systematics Austria
Scientific Council of the Convention on Migratory Species
Turtle Evolutionary Society
West Indian Ocean Marine Science Association
World Flora Online Council
World Register of Marine Species

Access to survey methods, data, and other resources on OSF public archive

All valid survey data, the survey, all analysis code and codebook, and all results are available on OSF at this link: https://osf.io/tz7ra/?view_only=4b1bc810ef794f7f9bb57240611989af

The survey pre-registration can be found here: https://doi.org/10.17605/OSF.IO/7DFWT

Files on OSF:

Survey_All_Languages.pdf

A printout of the survey from Qualtrics. Questions are provided in English, Spanish, French, German, Portuguese, and Simplified Chinese

Survey Methods and Data Availability.pdf

This document.

Survey Variable Codebook.csv

The survey codebook for survey pre-registration describing all variables.

Survey Variable Codebook 05.18.22.csv

The survey codebook that was adapted from the pre-registration version for final analysis.

Tax List Survey Valid Data_v7.xlsx

The version of the survey data used for all analysis.

Gov_Tax_Lists_Stata_Do_File.do

The Stata do file containing the code used for analysis.

STATA Do File_Analysis Code.pdf

A pdf version of the Stata .do file containing the code used for analysis.

Tabular Results.pdf

All outputs from Stata used in survey analysis.

Figures.pdf

Graphical figures of the analysis of results produced using Stata
